# Design of the PROstate cancer follow-up care in Secondary and Primary hEalth Care study (PROSPEC): a randomized controlled trial to evaluate the effectiveness of primary care-based follow-up of localized prostate cancer survivors

**DOI:** 10.1186/s12885-020-07112-9

**Published:** 2020-07-08

**Authors:** Barbara M. Wollersheim, Kristel M. van Asselt, Henk G. van der Poel, Henk C. P. M. van Weert, Michael Hauptmann, Valesca P. Retèl, Neil K. Aaronson, Lonneke V. van de Poll-Franse, Annelies H. Boekhout

**Affiliations:** 1 Division of Psychosocial Research and Epidemiology, The Netherlands Cancer Institute-Antoni van Leeuwenhoek Hospital, Plesmanlaan 121, 1066 Amsterdam, CX The Netherlands; 2 Department of General Practice, Amsterdam UMC location AMC, Amsterdam, The Netherlands; 3 Department of Urology, Antoni van Leeuwenhoek Hospital, The Netherlands Cancer Institute, Amsterdam, The Netherlands; 4 Institute of Biostatistics and Registry Research, Brandenburg Medical School, Neuruppin, Germany; 5 Department of Research, Netherlands Comprehensive Cancer organization (IKNL), Utrecht, The Netherlands; 6 Department of Medical and Clinical Psychology, CoRPS – Center of Research on Psychology in Somatic Diseases, Tilburg University, Tilburg, The Netherlands

**Keywords:** Prostate cancer, Survivorship, Follow-up, Primary care, Secondary care, General practitioner, Specialist, Randomized controlled trial

## Abstract

**Background:**

In its 2006 report, *From cancer patient to cancer survivor: lost in transition*, the U.S. Institute of Medicine raised the need for a more coordinated and comprehensive care model for cancer survivors. Given the ever increasing number of cancer survivors, in general, and prostate cancer survivors, in particular, there is a need for a more sustainable model of follow-up care. Currently, patients who have completed primary treatment for localized prostate cancer are often included in a specialist-based follow-up care program. General practitioners already play a key role in providing continuous and comprehensive health care. Studies in breast and colorectal cancer suggest that general practitioners could also consider to provide survivorship care in prostate cancer. However, empirical data are needed to determine whether follow-up care of localized prostate cancer survivors by the general practitioner is a feasible alternative.

**Methods:**

This multicenter, randomized, non-inferiority study will compare specialist-based (usual care) versus general practitioner-based (intervention) follow-up care of prostate cancer survivors who have completed primary treatment (prostatectomy or radiotherapy) for localized prostate cancer. Patients are being recruited from hospitals in the Netherlands, and randomly (1:1) allocated to specialist-based (*N* = 195) or general practitioner-based (*N* = 195) follow-up care. This trial will evaluate the effectiveness of primary care-based follow-up, in comparison to usual care, in terms of adherence to the prostate cancer surveillance guideline for the timing and frequency of prostate-specific antigen assessments, the time from a biochemical recurrence to retreatment decision-making, the management of treatment-related side effects, health-related quality of life, prostate cancer-related anxiety, continuity of care, and cost-effectiveness. The outcome measures will be assessed at randomization (≤6 months after treatment), and 12, 18, and 24 months after treatment.

**Discussion:**

This multicenter, prospective, randomized study will provide empirical evidence regarding the (cost-) effectiveness of specialist-based follow-up care compared to general practitioner-based follow-up care for localized prostate cancer survivors.

**Trial registration:**

Netherlands Trial Registry, Trial NL7068 (NTR7266). Prospectively registered on 11 June 2018

## Background

In 2006, the Institute of Medicine (IOM) published a report that described the health needs of cancer survivors [1]. This report concluded that the needs of cancer survivors are not being adequately addressed. That is, survivorship care should, in addition to surveillance for recurrent disease, manage late treatment effects, address comorbid conditions, attend to psychosocial needs, and promote healthy behaviors and lifestyle. Based on this report, the IOM recommended developing new healthcare pathways for cancer survivorship. An update in 2017 concluded that we have made progress in the past decade, but still improvements are necessary to ensure comprehensive and coordinated survivorship care [2].

In addition, cancer survivorship care is facing challenges due to an increase in cancer incidence, improved detection, and improved survival rates [3]. This is particularly true for prostate cancer. In 2018, almost 450.000 men were newly diagnosed with prostate cancer in Western Europe, accounting for 22% of all new cases of cancer in males [4]. In the United States, the estimated number of new patients with prostate cancer in 2019 was 174.650 [5]. Moreover, survival rates have increased by 30% in the last 30 years in both Western Europe and the United States, resulting in a substantial increase in the number of prostate cancer survivors (5-year survival rate of 88 and 98%, respectively) [5, 6]. In the United States, there are currently over 3 million prostate cancer survivors; in the Netherlands this is about 86.000 [5, 7].

Currently in most Western countries, patients who have completed primary treatment for localized prostate cancer are often included in a specialist-based follow-up care program at the hospital. Routine follow-up care for localized prostate cancer survivors includes periodic visits to test the prostate specific antigen (PSA) [8–10]. A detectable PSA level after prostatectomy is considered a biochemical recurrence (BCR). In clinical care, BCR can (in 16–35% of cases) trigger secondary therapy for prostate cancer, including salvage local treatment or androgen deprivation therapy (ADT) [11, 12]. Managing long-term and late treatment effects and providing psychosocial support to maintain health-related quality of life (HRQOL) are also important goals of survivorship care. After primary treatment, many prostate cancer survivors experience late effects of treatment, including urinary symptoms, bowel symptoms, sexual dysfunction, symptoms related to androgen ablation, and adverse psychosocial and relationship effects [13–19].

In order to address the needs of the growing population of prostate cancer survivors in the long-term, current survivorship care models are unsustainable [1]. Different models of follow-up care for cancer patients have been proposed. There is evidence that follow-up care for cancer patients can be provided by medical specialists, general practitioners (GPs), nurses, or by sharing the care among a multidisciplinary team [20–30]. Intervention studies for chronic diseases, such as diabetes or cardiovascular disease, suggest that it is possible to coordinate follow-up between primary- and secondary care providers [31–33]. GPs, who traditionally play a crucial role in providing continuous and comprehensive care for most patients with chronic diseases, could similarly consider the role of providing follow-up care to cancer survivors. National health councils of the United States, the United Kingdom, and the Netherlands have advised giving primary health care professionals a greater role in the follow-up of cancer survivors [1, 3, 34, 35]. This is supported by evidence from randomized controlled trials of patients with breast and colorectal cancer that have shown no significant differences in adverse outcomes for patients between primary versus secondary follow-up care [23, 25–27].

There is evidence that prostate cancer patients have increased their use of primary health care 3 to 5 years after cancer diagnosis [36, 37]. Importantly, almost half (48%) of all prostate cancer patients are aged 70 years or older [38], and often have other chronic health conditions [9, 39]. A small Australian study of a shared-care follow-up model (between GPs and hospitals) for prostate cancer survivors suggests it is feasible to implement PSA testing in primary care [24].

Currently, however, there is little empirical evidence on effectiveness of prostate cancer follow-up care in primary care versus secondary care. Most of the clinical trials conducted, to date, have focused on breast and colon cancer survivors or on shared care models [23–27, 40, 41], and have typically not included an assessment of psychological morbidity and quality of life [41].

### Objectives and hypotheses

This multicenter, randomized, non-inferiority trial, the PROSPEC study, is designed to compare the (cost-) effectiveness of a GP-based versus a specialist-based follow-up care program for localized prostate cancer survivors. We hypothesize that GP-based follow-up is as effective as specialist-based follow-up care in terms of (1) adherence to the prostate surveillance guideline regarding the timing and frequency of PSA measurements; (2) the time from a BCR to prostate cancer retreatment decision-making; (3) the management of treatment-related side effects, as experienced by patients; (4) health-related quality of life and prostate cancer-related anxiety; (5) continuity of care and; (6) costs-effectiveness.

## Methods/design

### Population and setting

Prostate cancer survivors who have completed primary treatment (prostatectomy or radiotherapy) for localized prostate cancer are being recruited from 12 hospitals across different regions in the Netherlands. Eligible patients are those diagnosed with invasive prostate cancer, stage cT1a–cT3; cN0–1, cM0, pNx–pN1; R0–1, who have had a prostatectomy or have completed radiotherapy (with or without androgen deprivation therapy (ADT)) as primary treatment, and are without evidence of recurrence (PSA < 0.1 ng/ml after prostatectomy or PSA < nadir+ 2.0 ng/mL after radiotherapy). Patients are excluded from the study if: they have not completed their primary treatment less than 6 months prior to randomization; are under active surveillance; are under investigation for possible recurrence; do not have a community-based GP to provide care; are actively followed by a cancer specialist for another primary cancer; are (previously) enrolled in a study requiring ongoing follow-up by a cancer specialist; have serious (treatment-related) toxicity that requires treatment; or are unable to understand the Dutch language; or do not provide written informed consent.

### Usual care- specialist based

Men in the usual care group will receive specialist-based follow-up care according to current hospital practice as outlined in Table 1, consistent with current Dutch and European prostate cancer surveillance guidelines [10, 42].

**Table 1:** Prostate cancer surveillance guideline [10, 42]

Surveillance	Year 1	Year 2–3	Year 4–10
Office visits	3, 6, and 12 months	Every 6 months	Annually
PSA monitoring	3, 6, and 12 months	Every 6 months	Annually
Physical examination	Only if indicated	Only if indicated	Only if indicated

### Intervention- primary care based

The intervention is based on a primary care model where localized prostate cancer survivors will be referred to their GP after the first follow-up visit at the hospital. In many western countries, as in the Netherlands, the GP is the first contact point for getting healthcare and the gatekeeper to secondary care. All patients will receive comparable follow-up care as the usual care group, as outlined in Table 1 [10, 42]. An overview of the guideline will be provided to the GP. In addition, information will be given to the GP about the patients’ primary cancer treatment, complications or treatment-related side effects (e.g. physical side effects like urinary incontinence, erectile problems, bowel problems and psychosocial problems) and the management of these side effects (e.g. pharmacological interventions, referral to a pelvic floor physiotherapist, referral to a psychologist, etc.). Information will also be given about the risk of recurrence, signs and symptoms of recurrence and recommended steps and procedures in the case of suspicion of recurrence. GPs will be asked to refer patients back to the hospital when the PSA level is > 0.2 ng/mL after surgery or > 2 ng/mL over nadir (i.e. the lowest PSA level) after radiotherapy in order to further evaluate the presence of a BCR.

### Randomization

In total, 390 consenting men will be randomized to either the GP-based (*n* = 195) or specialist-based (n = 195) follow-up care group. Randomization will be on a 1:1 ratio. The minimization technique will be applied using a randomization program (ALEA, FormVision, Abcoude, the Netherlands) to balance usual care with the intervention within a hospital on type of primary treatment (prostatectomy or radiotherapy) and clinical stage (according to the European Association of Urology (EAU) risk scores: low risk; intermediate risk; high risk [10]). Blinding of participants and clinicians is not possible due to the nature of the intervention.

### Recruitment

**Fig. 1:**
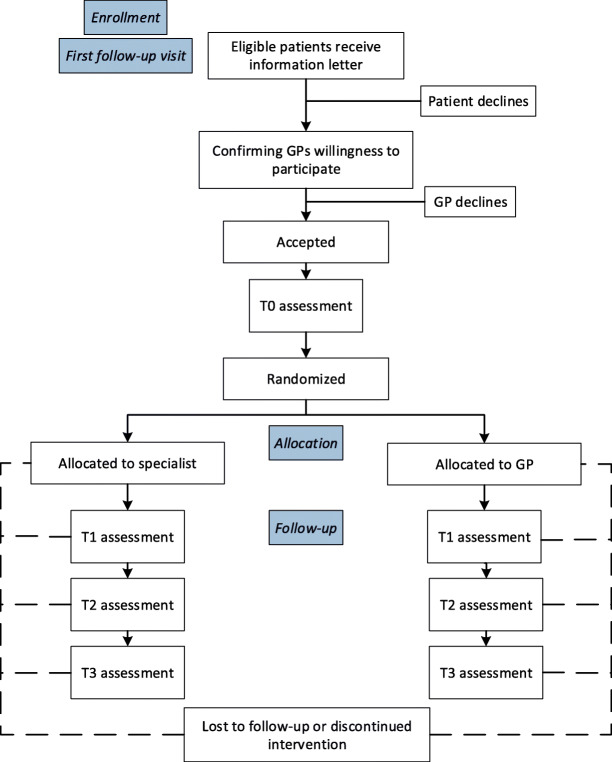
Study flow chart. First follow-up visit is ≤6 months post-treatment. T0 = measurement prior to randomization; T1 = measurement 12 months post-treatment, T2 = measurement 18 months post-treatment, T3 = measurement 24 months post-treatment. Abbreviations: GP = General Practitioner

We are recruiting patients from academic and general hospitals in the Netherlands. Medical specialists are asked to identify eligible patients at the first follow-up visit after primary treatment has been completed. Eligible patients are invited to participate in the study and, if interested, receive the information letter of the study. One week after the invitation, patients are contacted by telephone to explain the GP-based follow-up care and the RCT, and to confirm their willingness to participate in the study. After a patient has indicated his willingness to participate, his GP is contacted by telephone and asked whether (s)he is willing to participate in the study. If a GP declines participation, the patient cannot be included in the study and will be personally informed by telephone. Consenting patients will be randomized to specialist-based (usual care) or GP-based (intervention) follow-up care. Figure 1 details the study flow chart.

All participants will be followed during a 2-year study period. Patients allocated to GP-based follow-up may be referred back to the hospital at any time during the study. Similarly, patients allocated to specialist-based follow-up are free to consult their GP any time during the study. Patient recruitment and data collection for this trial started in July 2018.

### Data collection

Data are collected prior to randomization (T0), and at 12 (T1), 18 (T2), and 24 (T3) months after primary treatment has ended. The primary outcome is being abstracted from the medical records of participating hospitals and primary care practices. Secondary outcomes are also being abstracted from the medical records and collected using validated questionnaires. A reminder is sent to participants who do not return the questionnaire within 2 weeks. If a participant does not complete the questionnaire 2 weeks after the reminder, he will be contacted by telephone.

### Study outcomes

#### Primary study outcome

Adherence to the prostate cancer surveillance guideline is assessed on the basis of the timing and frequency of PSA measurements (Table 1) and will be assessed at T0, T1 and T3. A detailed description of the outcome measures are provided in Table 2.

**Table 2:** Outcome measures

Description of outcome	Assessment	Description of measure
Sociodemographic and clinical data	Sociodemographic data, disease and treatment characteristics will be abstracted from medical records or reported by the patient.	Patient reported: place of birth, marital status, educational level, employment, lifestyle factors (i.e. smoking, alcohol consumption, length and weight), and the self-administered comorbidity questionnaire [43].
		Medical records: birth-month and year, hospital where primary treatment took place, referred specialist, date of diagnosis, date and type of treatment, tumor characteristics (clinical and pathological stage).
**Primary outcome**		
Adherence to the prostate cancer surveillance guideline (Table 1)	PSA measurements	Number of PSA measurements will be abstracted from medical records.
**Secondary outcomes**		
The time from a BCR to prostate cancer retreatment decision-making	PSA value and referrals	The time from any detectable PSA level (> 0.2 ng/mL after surgery, > 2.0 ng/mL over nadir after radiotherapy) to the decision of prostate cancer retreatment in the hospital.
The management of treatment-related side effects	Assessment of Patients’ Experience of Cancer Care (APECC) survey [44]	37 items, organized into 10 scales in the following six areas: access to care; interaction with physicians; interaction with other members of the health care team; discussion of health promotion; perceptions of coordination of care; and the management of treatment-related side effects.
Health-related quality of life	EORTC Quality of Life Questionnaire Core 30 (QLQ-C30) [45]	30 items, organized into 5 functional scales (physical, role, emotional, cognitive, social), 3 symptom scales (pain, fatigue, and emesis), 6 items (dyspnea, sleep disturbance, appetite loss, constipation, diarrhea, and financial impact), and an overall QL scale.
Prostate cancer-related quality of life	EORTC Prostate cancer specific module (PR25) [46]	25 items, organized into 5 scales (urinary symptoms, bowel symptoms, hormonal treatment-related symptoms, sexual activity, and sexual functioning) and one item (incontinence aid).
Prostate cancer-related anxiety	Memorial Anxiety Scale of Prostate Cancer (MAX-PC) [47, 48]	18 items, organized into one scale consisting of 3 subscales (general prostate cancer anxiety, anxiety related to PSA levels in particular, and fear of recurrence).
Continuity of care	Nijmegen Continuity Questionnaire (NCQ) [49]	28 items, organized into one scale consisting of 3 subscales (personal continuity, care provider knows me and shows commitment, and team/cross-boundary continuity).
Cost-effectiveness	EuroQol 5-Dimension (EQ-5D-5L) [50]	5 items (dimensions) multi-attribute utility questionnaire that measures mobility, self-care, usual activities, pain/discomfort and anxiety/depression in 5 levels.
	Health care costs	Medical activities abstracted from the management systems of the hospitals and GP practices
	Indirect costs	Patient reported productivity losses [51], medical consumption [52] and travel costs

*Abbreviations*: *PSA* Prostate Specific Antigen, *BCR* Biochemical Recurrence, *EORTC* European Organization for Research and Treatment of Cancer, *QL* Quality of Life, *GP* General Practitioner

#### Secondary study outcomes

The time from a BCR to prostate cancer retreatment decision-making will be assessed at T0, T1 and T3. This is defined as the time from a rising PSA level after surgery (> 0.2 ng/mL) with or without radiotherapy or a rising PSA level of 2 ng/mL over the post-treatment nadir after radiotherapy to the decision regarding prostate cancer retreatment.

Other secondary outcome measures will assess the self-reported management of treatment-related side effects, health-related quality of life, prostate cancer-related quality of life, prostate cancer-related anxiety, and cost-effectiveness as described in Table 2. They will be assessed at T0, T1, T2, and T3 time points. Perceived continuity of care will be assessed in the T2 questionnaire.

#### Process evaluation

Alongside the RCT, we will conduct a process evaluation by interviewing patients, GPs, and specialists with the purpose of identifying barriers and facilitators of GP-based prostate cancer follow-up care. The methodology of this process evaluation will be described separately. If the results of the trial support the cost-effectiveness of GP follow-up care, the results of the process evaluation are expected to enable the transition of follow-up care to the GP.

### Power calculation

A total of 390 patients will be entered in this trial, 195 patients in each arm. Based on the objectives, the sample size is calculated separately for patients treated with prostatectomy (*n* = 270) and patients treated with radiotherapy (*n* = 120).

#### Adherence to the prostate cancer surveillance guideline and time from a BCR to prostate cancer retreatment decision making

With a sample of 270 patients who have been treated with prostatectomy, the prospective design will allow for testing of the main effect of GP-based versus specialist-based follow-up care on adherence to the prostate surveillance guideline, as represented by the timing and frequency of PSA assessments. It is expected that 90% of the patients in the usual care group will be assessed according to the guideline, defined as 4 PSA measurements in the study period of 2 years. This is in line with the observed adherence to PSA testing recommendations in a previous randomized controlled trial of shared care for follow-up of men with prostate cancer [24]. We consider an adherence percentage as low as 80% (10 percentage points non-inferiority margin) in the GP-based group as acceptable and lower adherence percentages as unacceptable. With 270 prostatectomy patients we have 86% power at a one-sided significance level of 5%.

We will also evaluate the difference between arms in time from a BCR to prostate cancer retreatment decision-making. Based on data of a retrospective cohort study of 1340 patients who were treated with prostatectomy at the Antoni van Leeuwenhoek Hospital and expert opinion, an acceptable mean time to decision-making is thought to be 30 days. We will evaluate whether the time to decision-making is substantially longer in the GP-arm than the specialist-based arm. We consider the maximally acceptable average time to decision-making in the GP-based arm as 90 days (60 days non-inferiority margin). In the absence of empirical data on the standard deviation (SD) for the time to decision-making, we assume an SD of 20 days in the specialist-arm and 60 days in the GP-arm. With 270 prostatectomy patients we expect a total of 7 patients with a BCR in each arm, which enables us to investigate the time from a BCR to prostate cancer retreatment decision making.

#### The management of treatment-related side effects as experienced by patients

With a sample of 120 patients who have been treated with radiotherapy, we are able to test the management of treatment-related side effects as experienced by patients. Hence, we will focus on questions from three scales of the “Assessment of Patients’ Experience of Cancer Care (APECC) survey” (information exchange; physicians’ affective behavior; and physicians’ knowledge) [44]. We will use the mean score of those three scales to measure patient satisfaction with the follow-up care. Arora and colleagues observed SDs between 15.8 and 24.9 for these three scales [44]. We assume that these SDs are generalizable to our hospital patient group. For a SD of 15.8, power is 80% to detect a clinically relevant difference of 8.1 points (effect size = 8.1/15.8 = .51) with 60 patients in each group (two-sided alpha 5%) [53].

#### Patient-reported outcomes

For the patient-reported outcomes (e.g. HRQOL, prostate-specific anxiety, etc.) we will compare changes in mean questionnaire scores over time between the usual care and the intervention group. Based on 390 patients and a survey attrition of 20%, power is at least 80% to detect a difference in questionnaire-based outcomes between patients who had their follow-up at their GP or specialist with an effect size of 0.1, at a two sided-significance level of 5%. With this sample size we will be able to detect clinically meaningful differences [53].

### Data analysis

#### Primary study outcomes

All analyses will be performed based on the intention-to-treat principle. Analyses will first be performed to evaluate the comparability of the groups at baseline on sociodemographic and clinical variables. The percentages of patients with four PSA tests in a period of 2 years will be compared in the two arms by using chi-square tests and multivariable by logistic regression.

#### Secondary study outcomes

For patients treated with prostatectomy, time from a BCR to prostate cancer retreatment decision-making will be evaluated by Kaplan-Meier methods including log-rank tests as well as multivariable Cox proportional hazards regression. Non-proportionality of hazards will be assessed by Schoenfeld residuals. In exploratory analyses, we will perform subgroup analyses by patient characteristics (e.g.: clinical stage: ≤ cT2b-c or cT3).

Scores for the APECC, EORTC QLQ-C30 and QLQ-PR25, MAX-PC, NCQ, EQ-5D-5L questionnaires will be calculated according to published scoring algorithms. Between-group differences over time in mean scores will be tested using a mixed effects modelling approach. For the moderation analysis, a regression based model will be constructed for each potential moderator separately in order to estimate the conditional (interaction) effect. Standardized effect sizes will be calculated by dividing the difference in mean change scores from baseline to follow-up between groups by the pooled baseline standard deviation.

The cost-effectiveness analysis will compare the costs and health benefits between both groups. This comparison is typically expressed in incremental cost, − health effects (often quality-adjusted life-years or QALYs), and - cost-effectiveness ratio (ICER) (e.g., incremental cost per QALY gained). A societal perspective from the Netherlands, plus lifelong time horizon will be adopted, according to the Dutch guidelines [54]. A trial-based analysis will be combined with a Markov decision analytic model, in order to capture both the trial endpoints at 2 years follow-up as well as the life long time horizon. Using a monthly cycle length, the model will simulate the lifelong course of events in a hypothetical cohort of prostate cancer survivors. We will include the estimation of the degree of uncertainty about each input parameter and the use of (probabilistic) sensitivity analyses. We will apply the most relevant severity-based ceiling ratio between €20,000 and €80,000 per QALY [55]. If necessary, Value of Information (VOI) analysis will be performed to support decision-making regarding adoption and further research [56].

### Ethical approval and consent to participate

The study received ethical approval from the institutional review board of the Antoni van Leeuwenhoek hospital, a specialized cancer center located in Amsterdam, the Netherlands (METC18.0033/M17PRO). The trial is registered in the Netherlands Trial Registry (NTR 7266). All patients will provide written informed consent before participating in the study.

### Safety reporting

An independent safety committee is formed to review safety-related data of patients participating in the PROSPEC study. The safety committee consists of an epidemiologist, a urologist and a GP. All of the committee members are independent of the study, and none has a conflict of interest with the sponsor of the study. During inclusion, the safety committee will meet twice: when 20 and 100 patients have finished the first 12 months of the intervention. The safety committee will advise the study investigators on the compliance to the prostate surveillance guideline, represented by the number of PSA measurements (primary endpoint), the time from a BCR to prostate cancer retreatment decision-making (main secondary endpoint), and the number of adverse events caused by following the study protocol.

## Discussion

In order to care for the needs of the increasing number of prostate cancer survivors, a more comprehensive and sustainable follow-up care model is necessary. GPs play an essential role in providing continuous and comprehensive care, and could consider the role of providing follow-up care to some cancer survivors. The evidence in the studies conducted, to date, have been limited, focusing on other cancer sites, or on shared follow-up care models. In the current study, we are evaluating a GP-based follow-up care model for men treated for localized prostate cancer (prostatectomy or radiotherapy).

It is hypothesized that GP-based follow-up care will not differ significantly with specialist-based follow-up care regarding the adherence to the prostate surveillance guideline regarding: the number of PSA measurements; the time from a BCR to prostate cancer retreatment decision-making; the management of treatment-related side effects as experienced by patients; health-related quality of life and prostate cancer-related anxiety; continuity of care and; and costs-effectiveness.

Several limitations of the trial should be noted. Prostate cancer patients will be followed up for a period of 2 years, meaning that longer-term evaluation of outcomes will not be possible. The time frame of the study is limited by the available research funding. We also recognize that not all medically eligible patients will be willing to be randomized to GP-based or specialist-based follow-up care, resulting in a study sample composed of men who have no strong preference in this regard. We will monitor the number of patients who decline to participate in the study, and the reasons for not participating, when possible.

This trial has several notable strengths, including its multicenter, randomized design, the use of an intention-to-treat strategy for the data analysis, and the inclusion of a cost-effectiveness analysis. The trial has a pragmatic character, as current practice is compared with GP-based care in the real-world healthcare system. Moreover, a process evaluation will be executed alongside this trial in order to understand and identify factors that influence GP-based follow-up care. If our trial indicates that a GP-based follow-up care is cost-effective, the process evaluation will help to describe the actual exposure to the intervention and to understand the barriers and facilitators to support effective implementation.

In conclusion, the PROSPEC trial will provide empirical evidence regarding the viability and effectiveness of a GP-based follow-up care program for localized prostate cancer patients. Especially within the current context of the rising number of prostate cancer survivors and the demands for a more comprehensive and sustainable follow-up care model, this type of research is of paramount importance as it can contribute to resolving some of the current challenges facing cancer survivorship care. If primary care-based follow-up for prostate cancer patients is feasible, the findings would also hopefully be of relevance to other groups of cancer survivors.

## Supplementary information

**Additional file 1.**

## Data Availability

The dataset used and analyzed during the current study will be available from the corresponding author (stored in a data repository at the Netherlands Cancer Institute) on reasonable request.

## Abbreviations

ADT: Androgen Deprivation Therapy
BCR: Biochemical Recurrence
GP: General Practitioner
HRQOL: Health-Related Quality of Life
PSA: Prostate Specific Antigen
