# Supplementary material for: Design of the PROstate cancer follow-up care in Secondary and Primary hEalth Care study (PROSPEC): a randomized controlled trial to evaluate the effectiveness of primary care-based follow-up of localized prostate cancer survivors
Source: BMC Cancer. 2020 Jul 8;20:635. doi: 10.1186/s12885-020-07112-9 (PMC7346492; doi:10.1186/s12885-020-07112-9)
Supplement: Supplementary file 1 — Additional file 1. [file 12885_2020_7112_MOESM1_ESM.rtf]

Subject information
NLxxxxx.xxx.xx – version [no.] [date]		page 1 of 26


Appendix 4: Informed Consent Form representative

Research about prostate cancer follow-up care in secondary and primary health care (PROSPEC study)

I have read the information letter. I was also able to ask questions. My questions have been answered sufficiently. I have had enough time to decide whether I want to participate in the study.

I understand that participation is voluntary. I also know that I can decide at any time that I would rather not participate. Without having to provide any reason.

I give consent to collecting and using my data to answer the research question in this study as described in the information letter.

I give consent to potentially be approached for a short interview about my experiences with my follow-up and this study.

I give consent to inform the general practitioner that I am participating in this study. I give consent to requesting information from the general practitioner. 

I know that for study monitoring purposes some individuals could have access to my data. These individuals are listed in this information letter. The storage of my data will be coded

I give consent to save my data for 15 years after completion of this study.

I agree to participate in this study.


Name of subject:
Signature:							Date: __ / __ / __

----------------------------------------------------------------------------------------------------------------
I hereby certify that I have informed the above person/persons fully about the said study.

If information becomes known during the study that could influence the consent of the legal representative, I will inform him/her of this on time.

Name of investigator (or his/her representative):
Signature:							Date: __ / __ / __

The representative will receive a complete information letter, along with a signed version of the informed consent form.
